# Hypophosphatemia: Unraveling a lethal connection with icu mortality in critically ill COVID-19 patients: a multicenter observational study

**DOI:** 10.5937/jomb0-52474

**Published:** 2025-03-21

**Authors:** Şahin Temel, Pervin Hanci, Akbudak Ismail Hakkı, Burçin Halaçli, Göksel Güven, Yeliz Bilir, Yüksel Recep Civan, Ezgi Özyilmaz, Neriman Defne Altintaş, Leyla Ferliçolak, Emre Aydin, Türkay Akbaş, Ali Ümit Esbah, Zuhal Güllü, Kamil Inci, Gülseren Elay, Arzu Topeli, Kürşat Gündoğan

**Affiliations:** 1 Erciyes University, School of Medicine, Department of Internal Medicine, Division of Intensive Care Medicine, Kayseri, Turkey; 2 Trakya University, School of Medicine, Department of Chest Diseases, Division of Intensive Care Medicine, Edirne, Turkey; 3 Pamukkale University, School of Medicine, Department of Internal Medicine, Division of Intensive Care Medicine, Denizli, Turkey; 4 Hacettepe University, School of Medicine, Department of Internal Medicine, Division of Intensive Care Medicine, Ankara, Turkey; 5 Ministry of Health, Istanbul Lütfi Kırdar Training and Research Hospital, Intensive Unit, Istanbul, Turkey; 6 Çukurova University School of Medicine, Department of Chest Diseases, Division of Intensive Care Medicine, Adana, Turkey; 7 Ankara University School of Medicine, Department of Internal Medicine, Division of Intensive Care Medicine, Ankara, Turkey; 8 Dicle University School of Medicine, Department of Internal Medicine, Division of Intensive Care Medicine, Diyarbakır, Turkey; 9 Düzce University School of Medicine, Department of Internal Medicine, Division of Intensive Care Medicine, Düzce, Turkey; 10 Ankara Yenimahalle Training and Research Hospital, Intensive Care Unit, Ministry of Health, Ankara, Turkey; 11 Gaziantep University, School of Medicine, Department of Internal Medicine, Division of Intensive Care Medicine, Gaziantep, Turkey

**Keywords:** SARS-CoV-2, hypophosphatemia, intensive care unit, respiratory failure, critical care, SARS-CoV-2, hipofosfatemija, jedinica intenzivne nege, respiratorna insuficijencija, intenzivna nega

## Abstract

**Background:**

Despite a lack of sufficient knowledge about the prevalence and impact of hypophosphatemia in critically ill COVID-19 patients, organ dysfunction, adverse clinical outcomes, and increased mortality have been consistently associated with hypophosphatemia across diverse patient populations. This retrospective, observational study aimed to investigate hypophosphatemia (HypoP) frequency and establish the correlation between variations in serum phosphorus levels and outcomes in critically ill patients with SARS-CoV-2.

**Methods:**

The research comprised 205 patients diagnosed with COVID-19 confirmed via RT-PCR. The study included COVID-19 patients who experienced respiratory failure and were in intensive care for more than 24 hours, and their phosphorus values were accurately documented. Clinical para meters, comorbidities, respiratory support requirements, and laboratory findings were analysed.

**Results:**

The study participants had a median age of 64 (IQR: 54-75 years), with hypertension being the most pre - valent chronic disease (46%). During the first three days of intensive care, 33% of the participants received conventional oxygen support, whereas 54% required intubation and mechanical ventilation (MV). During this period, hypophosphatemia was noted in 25% of patients, with an ICU admission median serum phosphorus level of 1.02 (0.87-1.25) mmol/L. The median duration of stay in the intensive care unit (ICU) was 7 days, significantly extended in patients with hypophosphatemia (p=0.046). Phosphorus levels on the third day of ICU stay were an independent predictor of ICU mortality. (COX, HR=1.48, 95% CI=1.11-1.98, p=0.006)

**Conclusions:**

During the first three days of ICU admission, 25% of SARS-CoV-2 critically ill adult patients presented with hypophosphatemia. This condition was found to increase ICU mortality rates and prolong ICU stays. Therefore, it is crucial to monitor serum phosphorus levels in the care of critically ill COVID-19 patients.

## Abstract (sr)

**Uvod:**

Uprkos nedostatku dovoljno znanja o prevalenciji i uticaju hipofosfatemije kod kritično obolelih pacijenata sa COVID-19, disfunkcija organa, nepovoljni klinički ishodi i povećana smrtnost dosledno se povezuju sa hipofosfatemijom u različitim populacijama pacijenata. Ova retrospektivna, opservaciona studija je imala za cilj da ispita učestalost hipofosfatemije (HypoP) i uspostavi korelaciju između varijacija nivoa fosfora u serumu i ishoda kod kritično obolelih pacijenata sa SARS-CoV-2.

**Metode:**

Istraživanje je obuhvatilo 205 pacijenata kojima je COVID-19 potvrđen RT-PCR testom. Studija je uključivala pacijente sa COVID-19 koji su imali respiratornu insuficijenciju i bili na intenzivnoj nezi duže od 24 sata, a čije su vrednosti fosfora bile precizno zabeležene. Analizirani su klinički parametri, komorbiditeti, potrebe za respiratornom podrškom i laboratorijski nalazi.

**Rezultati:**

Učesnici studije su imali median starosti od 64 godine (IQR: 54-75 godina), pri čemu je hipertenzija bila najzastupljenija hronična bolest (46%). Tokom prva tri dana boravka na intenzivnoj nezi, 33% učesnika je primilo konvencionalnu kiseoničku podršku, dok je u 54% slučajeva bila potrebna intubacija i mehanička ventilacija (MV). U tom periodu, hipofosfatemija je zabeležena kod 25% pacijenata, sa medianom nivoa fosfora u serumu pri prijemu u jedinicu intenzivne nege od 1,02 (0,87-1,25) mmol/L. Median trajanja boravka u jedinici intenzivne nege (ICU) je bio 7 dana, značajno duži kod pacijenata sa hipofosfatemijom (p=0,046). Nivoi fosfora trećeg dana boravka u intenzivnoj nezi su se pokazali kao nezavisan prediktor smrtnosti u ICU (COX, HR=1,48, 95% CI=1,11-1,98, p=0,006).

**Zaključak:**

Tokom prva tri dana boravka u jedinici intenzivne nege, hipofosfatemija je zabeležena kod 25% kritično obolelih odraslih pacijenata sa SARS-CoV-2. Utvrđeno je da ovo stanje povećava stopu smrtnosti u ICU i produžava trajanje boravka na intenzivnoj nezi. Stoga je praćenje nivoa fosfora u serumu kod kritično obolelih pacijenata od COVID-19 od ključnog značaja.

## Introduction

Hypophosphatemia has been recognised as a crucial factor linked to elevated mortality and morbidity in critically ill patients [1]. Phosphorus, an integral component of nucleic acids in DNA and RNA, is crucial in generating adenosine triphosphate, the energy source vital for all cellular functions [2]. Consequently, hypophosphatemia can manifest as mild symptoms like muscle weakness but can also lead to severe consequences, including respiratory failure and death [3].

The aetiology of hypophosphatemia in critically ill patients is multifaceted and may result from transcellular redistribution, characterised by decreased uptake and absorption, increased losses, or enhanced cellular phosphate uptake [4]. Recognised risk factors contributing to hypophosphatemia encompass anorexia, nutritional intolerance, pre-existing nutritional deficiencies, refeeding syndrome, blood gas disorders, and continuous haemodialysis [5].

### Objectives

Despite existing evidence indicating that hypophosphatemia prolongs hospitalisation and increases mortality in COVID-19 patients, there remains a dearth of comprehensive data on the frequency and repercussions of hypophosphatemia in this specific patient population [6] [7] [8]. This study aims to assess the frequency of hypophosphatemia, monitor serum phosphorus level changes, and investigate the relationship between hypophosphatemia and clinical outcomes in patients with SARS-CoV-2-related acute respiratory failure admitted to intensive care units.

## Materials and methods

This retrospective study was conducted in 21 intensive care units across 18 hospitals in Turkey. It serves as a secondary analysis of the study conducted by Gundogan et al. [9]. Patient enrollment occurred between 11 March 2020 and 11 June 2020.

The study involved patients diagnosed with SARS-CoV-2, confirmed by RT-PCR, who were admitted to the intensive care unit (ICU) due to respiratory failure lasting more than 24 hours. The study recorded the phosphorus values of these patients. Exclusion criteria included patients without recorded phosphorus values, those diagnosed with renal failure before, and those undergoing haemodialysis in the ICU.

Demographic information, age, gender, respiratory therapy, length of stay (LOS) in hospital and intensive care unit, APACHE-II score, laboratory findings (hemogram, blood urea nitrogen, creatinine, sodium, potassium, calcium, magnesium, albumin, ALT, AST, LDH, troponin, PT, Aptt, D-Dimer, ferritin, CRP, procalcitonin values), Sequential Organ Failure Assessment (SOFA) score at admission were recorded. Additionally, serum phosphorus levels during the first three days of ICU stay, cardiovascular complications, ICU-related events, and 28-day mortality were documented.

While nutritional status and diuretic use are recognised as potential confounding factors in the relationship between hypophosphatemia and ICU outcomes, data on these variables were not collected during the study.

Hypophosphatemia was defined as a phosphorus value <0.8 mmol/L during the first three days. Further categorisation included mild (0.8–0.65 mmol/L), moderate (0.64–0.32 mmol/L), and severe hypophosphatemia (<0.32 mmol/L) (10).

### Ethics

Approval for the study was granted by the Ministry of Health (2020-05-04T09_48_29) and the Erciyes University Ethics Committee (Date: 22 July 2020, No: 2020/401). The requirement for informed consent was waived.

### Statistical analysis

Statistical analysis was conducted using IBM SPSS Statistics Version 22. To determine normal distribution, Skewness, Kurtosis, Kolmogorov-Smirnov, and Shapiro-Wilk tests were employed. The Chi-Square or Fisher’s exact test was used to compare categorical variables. Descriptive statistics for categorical variables were provided. We used t-tests or Mann-Whitney U-tests as appropriate for group comparisons of numerical variables, with a statistical significance level of P<0.05. To identify the relationship between hypophosphatemia and mortality, we conducted a Cox proportional hazards regression analysis, yielding hazard ratios (HRs) and 95% confidence intervals (CIs).

## Results

**Figure 1:**
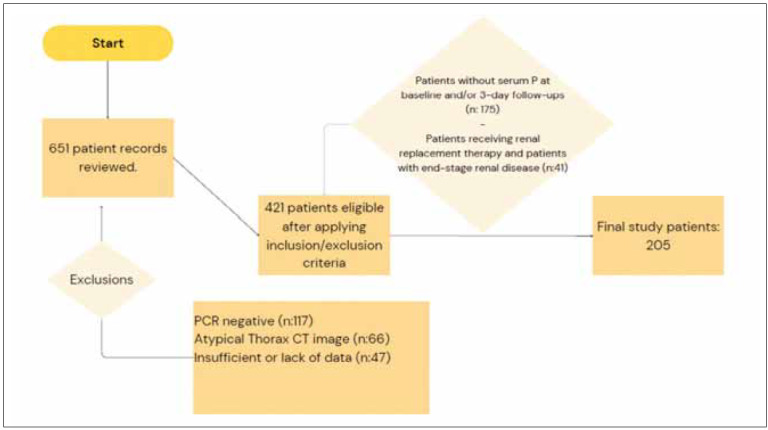
Flowchart Design: Patient Selection Process

In the TRICS-NET COVID-19 study, comprising 421 patients, 246 had serum phosphorus values, and after excluding those receiving haemodialysis and patients with end-stage renal failure, the study included 205 patients. (Figure 1, flow chart). The median age of the patients was 64 years (IQR: 54–75), with 61% being male. Hypertension and diabetes mellitus were the most prevalent comorbidities. Various respiratory support types were administered, including conventional O_2_ therapy (33%), high-flow nasal cannula O_2_ therapy (HFNCO, 10%), non-invasive mechanical ventilation (NIV, 3%), and invasive mechanical ventilation (IMV, 54%). Table 1

**Table 1:** Study subjects demographic and clinical characteristics.

Variables	Total<br>n: 205	Hypophosphatemia (+)<br>n: 51 (%25)	Hypophosphatemia (-)<br>n: 154 (%75)	p
**Age, year median (IQR)**	64 (54–75)	65 (55–74)	64 (53–75)	0.933
**Gender, n** (%)<br>Female<br>Male	80 (39)125 (61)	18 (35)33 (65)	62 (40)92 (60)	0.529
APACHE-II, median (IQR)	17 (12–27)	18 (15–28)	17 (12–26)	0.318
SOFA, median (IQR)	5 (3–8)	6 (3–8)	4 (3–8)	0.097
**Comorbidities, n (%)<br>**Hypertension<br>Diabetes mellitus<br>COPD<br>Malignancy<br>Heart Failure<br>CVD	<br>94 (46)<br>59 (29)<br>27 (13)<br>23 (11)<br>18 (9)<br>15 (7)	<br>19 (37)<br>13 (26)<br>6 (12)<br>6 (12)<br>2 (4)<br>3 (6)	<br>75 (49)<br>46 (30)<br>21(14)<br>17 (11)<br>16(10)<br>12 (8)	<br>0.155<br>0.549<br>0.732<br>0.887<br>0.157<br>0.650
**Respiratory support<br>(baseline and follow-up)<br>n (%)**<br>Standard O2<br>HFNCO<br>NIVM<br>IVM	<br>67 (33)<br>20 (10)<br>8 (3)<br>109 (54)	<br>12 (22)<br>4 (6)<br>3 (4)<br>34 (68)	<br>55 (36)<br>16 (11)<br>5 (3)<br>75 (50)	<br>0.921<br>0.308<br>0.106<br>**0.026**
**Covid-19 associated<br>cardiovascular event, n (%)**	11 (5.4)	4 (7.8)	7 (4.5)	0.365
Duration of IVM (days),<br>median (IQR)	7 (3–17)	7 (3–12)	8 (3–18)	0.570
Length of ICU stay, median<br>(IQR), day	7 (3–16)	10 (5–18)	6 (3–16)	**0.046**
Length of hospital stay,<br>median (IQR) day	17 (10–26)	18 (9–28)	17 (11–27)	0.929
ICU mortality, n (%)	73 (36)	21 (41)	52 (34)	0.338
28-day mortality, n (%)	75 (37)	20 (39)	55 (36)	0.653
Hospital mortality, n (%)	86 (42)	22 (44)	64 (42)	0.761

Cardiovascular complications (arrhythmia, myocardial infarction, myocarditis, and sudden cardiac arrest) were more common in patients with hypophosphatemia (7.8% vs 4.5%), although the difference was not statistically significant (p=0.36).

The median ICU length of stay was 7 days (IQR: 3–16), and it was significantly higher in the hypophosphatemia group (10 days, IQR: 5–18) compared to the non-hypophosphatemia group (6 days, IQR: 3–16) (p=0.046).

There was no statistical difference in the median hospital length of stay between the two groups (p=0.929).

The 28-day mortality rate of the patients was 42%, and there was no statistically significant difference between the groups (p=0.653).

Hypophosphatemia was present in 25% (n: 51) of patients.

**Figure 2:**
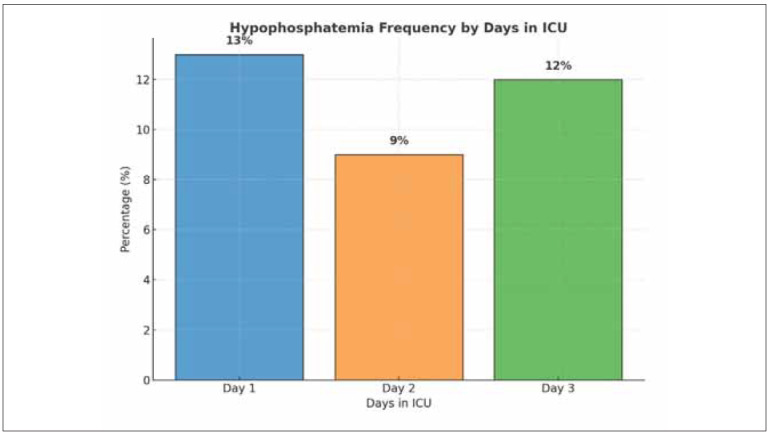
Frequency of hypophosphatemia three-day follow-up period.

**Figure 3:**
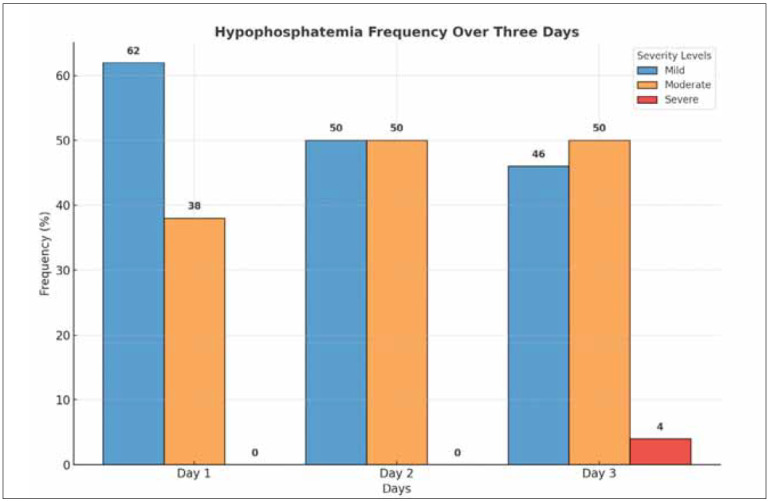
Frequency of mild, moderate, and severe hypophosphatemia over three days.

The distribution of hypophosphatemia on the first, second, and third days of ICU stay was 13%, 9%, and 12%, respectively. Figure 2 Figure 3 Table 2

**Table 2:** Study participants’ laboratory parameters.

Variables (admission)	Total	Hypophosphatemia (+)<br>n:51	Hypophosphatemia (-)<br>n:154	P
Phosphorus	1.02 (0.87–1.25)	0.77 (0.66–1.03)	1.08 (0.96–1.29)	0.000
WBCs (×10^9^/L), median (IQR)	8.3 (5.78–11.26)	7.7 (5.60–10.74)	9.23 (5.77–11.60)	0.241
Neutrophils (×10^9^/L), median (IQR)	6.5 (4.20–9.40)	6.15 (4.20–9.40)	7.20 (3.84–9.60)	0.586
Lymphocytes (×10^9^/L), median (IQR)	0.88 (0.59–1.22)	0.75 (0.54–1.10)	0.85 (0.50–1.33)	0.194
Hemoglobin (g/L), median (IQR)	12.40 (10.80–13.95)	12.10 (10.85–13.40)	12.10 (10.60–13.90)	0.717
Platelets (×10^9^/L), median (IQR)	207 (151–268)	210 (157–264)	194 (136–252)	0.476
Glucose (mg/dL), median (IQR)	126 (99–164)	140 (108–171)	118 (92–152)	0.173
BUN (mg/dL), median (IQR)	22 (13–37)	20 (12–33)	23 (14–47)	0.154
Creatinin (mg/dL), median (IQR)	0.85 (0.66–1.10)	0.89 (0.68–1.21)	0.99 (0.70–1.49)	0.058
Sodium (mmol/L), median (IQR)	137 (134–140)	137 (133–139)	137 (133–139)	0.058
Potasium (mmol/L), median (IQR)	4.04 (3.64–4.40)	3.83 (3.59–4.35)	4.12 (3.80–4.40)	0.082
Calcium (mg/dL), median (IQR)	8.30 (7.99–8.67)	3.83 (3.59–4.35)	8.30 (7.93–8.70)	0.857
Albumin (g/dL), median (IQR)	3.12 (2.75–3.50)	8.20 (7.90–8.60)	3.10 (2.60–3.40)	0.479
ALT (U/L), median (IQR)	3.12 (2.75–3.50)	3.17 (3.88–3.43)	25 (16–45)	0.811
AST (U/L), median (IQR)	26 (16–49)	26 (17–48)	36 (28–56)	0.883
Total bilirubin (mg/dL), median (IQR)	38 (26–65)	39 (31–56)	0.59 (0.40–0.81)	0.477
LDH (U/L), median (IQR)	0.59 (0.40–0.81)	0.50 (0.40–0.80)	395 (269–538)	0.508
Troponin (ng/L), median (IQR)	6.40 (0.10–28.85)	5.20 (0.04–19.80)	4.80 (0.04–28.80)	0.790
D-dimer (ng/mL), median (IQR)	1105 (564–2862)	1119 (665–2390)	1505 (630–3200)	0.664
Ferritin (ng/mL), median (IQR)	462 (184–899)	432 (203–787)	580 (151–970)	0.977
CRP (mg/L), median (IQR)	104 (46–167)	119 (66–179)	94 (53–152)	0.764
Procalsitonin (ng/mL), median (IQR)	0.26 (0.10–0.90)	0.34 (0.13–0.84)	0.34 (0.13–1.87)	0.562

Categorisation of hypophosphatemia based on serum phosphorus level revealed that mild hypophosphatemia was the most common.

In patients with hypophosphatemia, there was a higher requirement for IMV compared to the group without hypophosphatemia (68% vs 50%, p=0.026).

The groups did not differ in terms of various laboratory parameters, including hemogram, serum glucose, BUN, creatinine, sodium, potassium, calcium, albumin, ALT, AST, total bilirubin, LDH, troponin, ddimer, ferritin, CRP, and procalcitonin levels.

**Figure 4:**
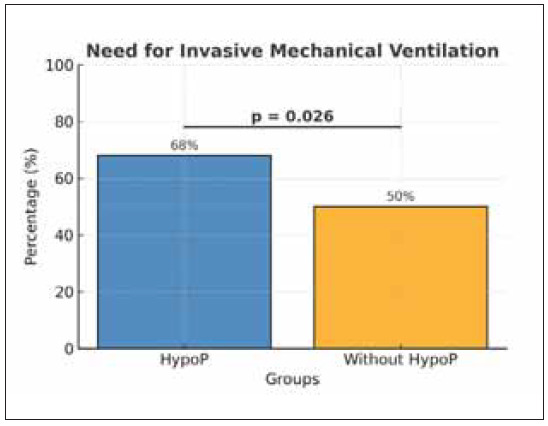
Need for invasive mechanical ventilation between groups.

Intensive care mortality for all patients was 36%. Mortality was 41% in the hypophosphataemia group and 34% in the other group, but there was no statistical difference (p=0.338). Table 3 Figure 4

**Table 3:** Cox proportional hazard analysis for evaluating the impact of phosphorus levels on ICU mortality. * Adjusted for age and gender

	HR (95% CI)	p
**Crude<br>Phosphorus level<br>(per 1 mg/dL decrease) **		
**Day 1<br>Day 2<br>Day 3**	0.75 (0.51–1.09)<br>1.08 (0.72–1.61)<br>1.49 (1.12–1.99)	0.131<br>0.700<br>0.006
Adjusted *<br>Phosphorus level<br>(per 1 mg/dL decrease)		
**Day 1<br>Day 2<br>Day 3**	0.98 (0.96–1.08)<br>1.20 (0.80–1.82)<br>1.46 (1.08–1.97)	0.188<br>0.381<br>0.014

Phosphorus levels on the third day of ICU stay were an independent predictor of ICU mortality (COX, HR=1.49, 95% CI=1.12–1.99, p=0.006). Even after accounting for confounding variables such as age and gender, the association between the phosphorus level on the third day and ICU mortality persisted (HR [95% CI] 1.46 [1.08–1.97], p=0.014).

In both the crude and adjusted models, there was no association between phosphorus levels on the first and second day and ICU mortality (p=0.131 and p=0.700 for the crude model, p=0.188 and p=0.381 for the adjusted model, respectively).

These findings highlight the prevalence of hypophosphatemia in critically ill SARS-CoV-2 patients and its association with specific clinical outcomes, including respiratory support requirements and ICU mortality. The study provides valuable insights into the relationship between hypophosphatemia and adverse outcomes in this patient population.

## Discussion

During the initial 3 days of follow-up, 25% of critically ill patients with COVID-19 exhibited hypophosphatemia. This prevalence is comparable to other studies conducted in 2013 and 2020, with rates ranging from 20% to 25%, but higher than the point prevalence study by M.M. Berger et al. [10] in 2020, which reported 15.4%. Our study exclusively focused on critically ill SARS-CoV-2 patients. Hypophosphatemia was recorded at any time during the 3-day follow-up, which may explain the variance in prevalence [11] [12] [13]. Factors such as nutritional intolerance, pre-existing nutritional deficiencies, refeeding hypophosphatemia, subcutaneous diabetes treatments, blood gas disorders, and losses due to diuretics and antacids may contribute to the higher prevalence observed.

The increased demand for adenosine triphosphate (ATP) by immune system cells fighting the COVID-19 infection can be attributed to the relationship between hypophosphatemia and respiratory failure in critically ill patients [13,14]. The subsequent decrease in phosphorus, a key component in ATP synthesis and regeneration, may lead to respiratory muscle weakness and, ultimately, respiratory failure [15]. This is supported by our findings, which showed a higher requirement for invasive mechanical ventilation (IMV) in the hypophosphatemia group compared to the non-hypophosphatemia group (68% vs 50%, p=0.02). A study also found an association between hypophosphatemia and respiratory failure, emphasising the need for mechanical ventilation [15,16]. Additionally, a study by Broman et al. [17] revealed that patients with hypophosphatemia had a longer duration of mechanical ventilation than the control group. Furthermore, hypophosphatemia was identified as a standalone predictor for weaning failure (OR: 0.43, 95% CI: 0.21–0.88, p=0.02) [18].

The study showed that patients with hypophosphatemia had a more extended stay in the ICU than those without hypophosphatemia (10 days vs 6 days, p=0.046). This is consistent with the results of two meta-analyses, which reported longer ICU stays for patients with hypophosphatemia. [19] [20]. The extended ICU stay in the hypophosphatemia group in our study may be indicative of poor clinical outcomes associated with hypophosphatemia.

The study found that ICU mortality was 25%. In this study, phosphorus levels were an independent predictor of ICU mortality on the third day after ICU admission. After adjusting variables such as age and gender, the association between phosphorus levels on the 3rd day and ICU mortality remained significant. Several studies have highlighted the critical role of phosphorus levels in predicting ICU outcomes, particularly mortality. Our findings, which demonstrate that third-day phosphorus levels independently predict ICU mortality, align with previous research. For instance, studies by Berger et al. [10] and Broman et al. [17] emphasise that sustained hypophosphatemia is associated with prolonged mechanical ventilation and higher mortality rates. The observed persistence of this association, even after adjusting for age, gender, and disease severity, underscores the clinical importance of monitoring and addressing phosphorus levels in critically ill patients. Integrating these findings into routine ICU practice could help refine prognostic models and guide early interventions to mitigate hypophosphatemia-related complications [10] [17] [21]. Despite lower APACHE-II scores in our study compared to that study, suggesting a potentially lower severity, ICU mortality in COVID-19 varies widely in the literature, ranging from 28% to 54% in different studies [22] [23] [24].

The heightened intensive care unit mortality within the hypophosphatemia group, juxtaposed with the well-established link between hypophosphatemia and respiratory failure, suggests a conceivable association pointing towards increased mortality in ICU patients with COVID-19. However, no statistically significant difference in 28-day mortality was observed between the study groups. A study from China reported a 28-day mortality of 39%, which was higher than our findings and may again be attributed to hypophosphatemia [25].

Strengths of our study include the continuous monitoring of phosphorus values during the 3-day follow-up in critically ill COVID-19 patients.

### Limitations

Nutritional interventions, phosphorus supplementation, diuretic use, and renal replacement therapies (RRT) significantly impact serum phosphorus levels and related clinical outcomes. However, data on these variables were not collected in this study, which represents a limitation in thoroughly assessing their potential influence. Nutritional deficiencies or refeeding syndrome may exacerbate hypophosphatemia by depleting serum phosphorus, impairing energy metabolism and muscle function. Diuretic use, particularly loop diuretics, increases renal phosphorus excretion, while RRT can lead to rapid phosphorus depletion during filtration processes, potentially worsening critical illness outcomes. Although these variables were not recorded, their absence should be considered when interpreting the findings, as they may have influenced the observed associations between hypophosphatemia and ICU outcomes.

## Conclusions

Our study has highlighted the association between hypophosphatemia at ICU admission and during the initial 3 days with increased mortality, the need for IMV, and extended LOS in critically ill SARS-CoV-2 patients. Ongoing surveillance of phosphorus levels, coupled with a comprehensive understanding of its origins and potential complications, remains essential in the care and treatment of critically ill COVID-19 patients. Further investigation is necessary to determine whether prevention or treatment of hypophosphatemia mitigates its adverse consequences.

## Dodatak

### Financial disclosure

There is nothing to declare.

### Conflict of interest statement

All the authors declare that they have no conflict of interest in this work.
